# Which Factors Are Associated with Monitoring Goal Progress?

**DOI:** 10.3389/fpsyg.2017.00434

**Published:** 2017-03-24

**Authors:** Betty P. I. Chang, Thomas L. Webb, Yael Benn, Chris B. Stride

**Affiliations:** ^1^Department of Psychology and Educational Sciences, Université Libre de BruxellesBrussels, Belgium; ^2^Department of Psychology, University of SheffieldSheffield, UK; ^3^Department of Psychology, Manchester Metropolitan UniversityManchester, UK; ^4^Management School, University of SheffieldSheffield, UK

**Keywords:** goal progress, monitoring, self-monitoring, evaluation, self-regulation, the ostrich problem

## Abstract

Three studies examined how people assess their progress on personal goals (e.g., whether they compare their progress to the past and/or to a desired target state), along with factors that might influence the nature of progress monitoring (e.g., whether the goal involves attaining a positive outcome or avoiding a negative outcome). Study 1 involved semi-structured interviews with 40 participants, in which we examined how participants monitored their progress and whether this was related to: (a) their level of self-efficacy, (b) whether the goal was prevention focused, and (c) whether goal progress was represented in quantifiable terms. Studies 2 (*N* = 492) and 3 (*N* = 481) were conducted online and additionally examined whether how participants monitored their progress differed as a function of the domain of the goal (i.e., whether it was related to physical development/health, finances, work/study, or social relationships). The findings suggest that participants: (i) were less likely to monitor their progress toward goals that were related to avoiding negative outcomes, (ii) were less likely to monitor their progress toward goals related to finances, work, or study with reference to the past, than progress toward other goals (e.g., those relating to physical development and health), (iii) found it easier to monitor their progress toward goals that they felt confident of attaining, but harder to monitor their progress toward goals related to work or study. Finally, the more participants thought about their goal in quantifiable terms, the more likely they were to monitor their progress, and the easier they found monitoring their progress to be. Taken together, these studies begin to describe the nature of progress monitoring and the factors that influence this important self-regulatory process.

## Introduction

People often want to know how they are progressing on their personal goals. For example, an employee with the goal to succeed at work might ask her boss for feedback, reflect on whether she is performing better than before, and keep track of whether her deadlines are being met. Theories of goal pursuit such as Control Theory (Carver and Scheier, 1981, 1982), Social Cognitive Theory (Bandura, 1977), and Goal Theory (Locke and Latham, 1990), suggest that people assess their goal progress by noting the attributes of their behavior or associated outcomes and comparing these with salient reference values (Carver and Scheier, 1990). Many studies have shown that monitoring progress helps people to achieve their goals (e.g., Bandura and Cervone, 1983; Schunk, 1983; Renn and Fedor, 2001; Harkin et al., 2016). However, despite the theoretical and practical importance of monitoring, little research has investigated how people monitor their goal progress, the likelihood of progress monitoring and the factors that influence this, despite evidence that doing so can inform interventions designed to encourage the use of monitoring, with a view to promoting goal attainment (e.g., Verhoeven et al., 2014; for a review, see Harkin et al., 2016). The present research, therefore, sought to identify how people monitor their goal progress, along with the factors that influence whether and how people do so, and how difficult they find monitoring their progress to be.

Monitoring goal progress involves comparing the current state with a reference value (Carver and Scheier, 1982, 1990). This reference value may be a desired target or goal (e.g., to weigh 55 kg) or a reference value in the past (e.g., weight last year); with the latter being known as a temporal comparison (Albert, 1977). While certain types of motivations may affect the reference values against which people evaluate their progress (e.g., the motivation to self-enhance may make people more likely to make temporal comparisons, since comparing current performance to the past tends to highlight improvement, Zell and Alicke, 2009), it is not yet clear whether other individual- and goal-related factors also influence the type of reference value that people adopt. Therefore, one aim of the present research is to identify other factors that influence the choice of reference value. We also investigate whether these factors influence the frequency with which people monitor their progress, as it has been demonstrated that more frequent progress monitoring can increase the likelihood that people will achieve their goal (e.g., Harkin et al., 2016). Furthermore, we examine whether these factors influence how easy monitoring goal progress is perceived to be; not least because people may avoid monitoring their progress when doing so is perceived as difficult (Liberman and Dar, 2009; Webb et al., 2013).

### What Factors Influence How People Monitor Their Goal Progress?

One factor that may play a role in monitoring goal progress is whether people think about their progress in quantifiable terms, such as how much weight has been lost (where the goal is losing weight), or how many essays have been marked (where the goal is marking a set of exam papers). Progress on some goals, such as having a rewarding relationship, may be more difficult to represent in quantifiable terms. Evidence suggests that people have a tendency to judge their performance based on quantifiable information, even in cases where that information is not diagnostic of the characteristic that they are evaluating (e.g., counting the number of pages read, when the goal is understanding, Liberman and Dar, 2009). According to Josephs et al. (1994), this ‘quantity principle’ is adopted because people favor information that is computationally simple (e.g., Hunt and Agnoli, 1991), available (Tversky and Kahneman, 1973), representative (Kahneman and Tversky, 1973), and attracts attention (Taylor and Fiske, 1978). Therefore, we predict that people will be more likely to monitor their goal progress if they feel able to assess it in quantifiable terms.

How people relate to their goals may also influence whether and how they monitor their goal progress. One important variable in this regard might be levels of self-efficacy, which reflect a person’s belief in their ability to achieve a goal (Bandura, 1977). For example, Bouffard-Bouchard et al. (1991) found that students with high levels of self-efficacy spent more time monitoring how long they spent working on a problem-solving task than those with low self-efficacy. This led us to predict that levels of self-efficacy would be positively associated with the frequency with which people monitor their goal progress.

A person’s motivation for achieving a goal or performing goal-related activities may also influence the reference value that they use and the extent of progress monitoring. Liberman and Dar (2009) propose that people may monitor their progress toward goals that have a prevention focus (i.e., goals that are motivated by security, responsibility, and/or the avoidance of losses; Higgins, 1997, 1998) more frequently, because such goals tend to require more immediate action than goals with a promotion focus (i.e., goals that are motivated by gains, Higgins, 1997, 1998; Freitas et al., 2002; Pennington and Roese, 2003). For example, Freitas et al. (2002, Experiment 2) found that people elected to start working on an application for a fellowship sooner if their attention was drawn to avoiding rejection (a prevention focus) rather than to being accepted (a promotion focus). However, Liberman and Dar (2009) also note that prevention-focused goals tend not to have an end state, because they are often concerned with avoiding a particular outcome, which may make it harder to assess progress. Thus, we also investigated whether people are more or less likely to monitor their progress toward goals with a stronger prevention focus.

Liberman and Dar (2009) also suggest that it may be more difficult for people to monitor their progress toward goals that are based on intrinsic motivation (i.e., those that fulfill needs related to competence, relatedness to others, and autonomy; Ryan and Deci, 2000) because activities that are intrinsically motivated are performed for their inherent satisfaction, rather than to attain some separable consequence. For example, a person may have the goal to spend time with friends simply because they enjoy socializing. In this case, there is no concrete outcome that the person is trying to achieve (other than perhaps some sort of feeling state), and thus it may be difficult to monitor the outcome of that goal. Therefore, we hypothesize that people will consider goals that are based on intrinsic motivation to be more difficult to monitor and, as a consequence, they may monitor their progress toward such goals less frequently.

Lastly, we examined whether the amount of time that people had spent pursuing their goal influenced whether they evaluated their goal progress with respect to a target or to a past state. This is because the findings of Bonezzi et al. (2011) suggest that, to the extent that the duration of goal pursuit is typically associated with being closer to achieving a goal, people who have worked on their goal for longer are likely to evaluate their progress with respect to a target end-state, while people who have just started striving for a goal are likely to evaluate their progress with respect to the past (or starting) state.

### The Present Research

Although progress monitoring is a central component of frameworks for understanding self-regulation, relatively little research has investigated the frequency with which people monitor their progress on their personal goals and how they assess their progress. Thus, we conducted three studies to investigate these questions. Study 1 interviewed participants and asked them how they assess their progress on their personal goals. This open-ended, exploratory method allowed us to understand how participants interpreted the constructs that we were interested in and helped to identify factors that might influence progress monitoring that, as yet, remain untested in the existing literature. In Study 2, participants completed questionnaires that measured how they assessed their progress toward one of their personal goals. Participants then evaluated their goal on the focal dimensions derived from Study 1 and rated the perceived instrumentality of their behavior. Study 2 also extended Study 1 to examine whether the domain of goal striving (e.g., health or work) influenced how participants monitored their progress. Finally, Study 3 provided a conceptual replication of Study 2 using multi-item measures.

## Study 1

### Method

#### Participants

Participants were recruited via an email sent to a list of staff and postgraduate volunteers at a large university in northern England. Forty people (*M*_age_ = 32.30 years, *SD* = 8.74, 28 females) responded and were paid £10 for their participation. Ethical approval for all studies presented in this paper was obtained from the University of Sheffield’s Department of Psychology Ethics Committee.

#### Procedure

Participants gave informed consent to take part in a semi-structured interview where they described up to five goals that they were currently working on. A series of open-ended and Likert-style questions was used to assess each of the variables pertaining to our hypotheses. The interviews ranged from 19 to 90 min, and were recorded and then transcribed. The first author and a second coder who was blind to the hypotheses coded participants’ responses to the open-ended questions. Inter-rater reliability was good (free-marginal kappa = 0.81 or higher) and disagreements were jointly resolved through discussion.

#### Measures

##### Nature of elicited goals

Participants were asked how long they had worked on each nominated goal for (in weeks), and why they were pursuing the goal. Responses to the latter question were used to identify whether the goal was: (a) prevention focused, and (b) based on intrinsic motivation. Prevention focused goals were coded as those based on security, responsibility, and/or the avoidance of negative outcomes (e.g., giving up smoking because it is unhealthy and expensive). Goals were coded as being intrinsically motivated if the person enjoyed performing the activity, and/or because it fulfilled their need for autonomy, competence, and relatedness.

##### Nature of progress monitoring

Participants were then asked questions about how they monitored their progress toward the target goal. First, they were asked “Do you have an idea of whether you are making progress toward this goal?” Those answering in the affirmative (all but one case) were then asked “How do you know that you are/are not making progress toward this goal?” Based on each participants’ responses to this question and how they described their goal, we coded whether they represented their progress in quantifiable terms (coded 1; e.g., in terms of weight lost, money saved, hours of time spent performing an activity or behavior) or not (coded 0; e.g., in terms of resolving psychological issues).

Participants were then asked: “When people think about their goal progress, sometimes they compare their current situation to the past and sometimes they compare it to a target that they want to meet. So when you think about your progress toward [your goal] do you compare your progress to how you used to [perform on that goal/your previous state], and/or how you would like to [perform on that goal/your desired state]?” Responses to this question were coded as those that involve a comparison to the past (e.g., “I compare my current weight with how much I used to weigh”), and/or those that involve a comparison to a target (e.g., “I compare my fitness to what I would like it to be”). Finally, participants rated how difficult they found monitoring their goal progress (on a 5-point scale anchored by 1 = very easy and 5 = very difficult) and, as a measure of self-efficacy, how confident they were that they could achieve the goal (1 = not at all confident to 5 = very confident).

### Results

#### Nature of Elicited Goals

Participants described 137 goals in total. There was considerable variation in how long participants had worked on their goals for (*M* = 141.80 days, *SD* = 315.28). Participants reported goals for which progress could be easily quantified (49.6% of goals) and goals for which progress could not be easily quantified (50.4%). About half of the goals were prevention focused, and about a quarter of the goals were based on intrinsic motivation (28.5%). Participants felt moderate levels of self-efficacy (*M* = 3.59, *SD* = 1.06). The range of goals that were nominated is presented in the Appendix.

#### Nature of Progress Monitoring

Participants sometimes compared their goal progress to a past state (63.5% of goals) and sometimes to a desired target (48.9% of goals). Participants considered monitoring goal progress to be moderately difficult (*M* = 2.42, *SD* = 1.21).

#### Factors Associated with the Nature of Progress Monitoring

We next examined which factors were associated with the nature of progress monitoring. Because participants could describe more than one personal goal, the data was structured with goals treated as distinct observations (i.e., ‘nested’) within each participant. Two multilevel logistic regression analyses tested the unique relationships between each predictor (e.g., characteristics of the focal goal) and the outcome variables (e.g., comparisons to the target or past). A multilevel linear regression with participants’ rating of the difficulty of monitoring progress as a continuous outcome variable was also conducted with the same predictors. **Table 1** summarizes the findings.

**Table 1:** Unstandardized relationships (B coefficients, SEs) between predictors (rows) and the nature of progress monitoring (columns) in Study 1.

	Comparison to the past^†^	Comparison to a target^†^	Difficulty of monitoring
Weeks spent on goal^a^	0.11 (0.26)	–0.28 (0.29)	0.05 (0.14)
Prevention focus	0.13 (0.39)	–0.96^∗^ (0.43)	0.30 (0.20)
Intrinsic motivation	–0.03 (0.41)	–0.76 (0.43)	0.08 (0.21)
Quantifiability	0.74 (0.42)	0.88^∗^ (0.45)	–0.59^∗∗^ (0.21)
Self-efficacy	0.05 (0.18)	–0.33 (0.20)	–0.24^∗^ (0.10)

*^∗^*p* ≤ 0.05, ^∗∗^*p* < 0.01. ^†^Logit transformed binary DVs. For these DVs the odds ratio for each predictor’s effect the odds ratio can be calculated by taking the exponential of the corresponding *B* coefficient reported in the table. ^a^This variable was log transformed prior to analysis.*

None of the factors were significantly associated with the likelihood that participants reported assessing their progress with respect to the past (the closest being whether progress was thought of in quantifiable terms, B = 0.74, *SE* = 0.42, *z* = 1.77, *p* = 0.08). Participants were less likely to assess their goals with reference to a target if their goal was related to security and responsibility (B = -0.96, *SE* = 0.43, *z* = -2.24, *p* = 0.03, *odds ratio* = 0.38) than if it was not. Progress toward goals that were thought of in quantifiable terms were more likely to be compared to a target (B = 0.88, *SE* = 0.45, *z* = 1.98, *p* = 0.05, *odds ratio* = 2.41) than progress toward goals that were not thought of in quantifiable terms. Participants also reported that it was easier to assess their progress on goals for which progress was quantified [*B* = -0.49, *SE* = 0.21, *t*(128.80) = 7.83, *p <* 0.01, B = -0.59] and when they felt confident of achieving their goal (i.e., had high self-efficacy) [*B* = -0.20, *SE* = 0.10, *t*(114.44) = -2.47, *p* = 0.02, B = -0.24].

### Discussion

Study 1 used an open-ended, exploratory approach to investigate whether and how people monitor their progress toward goals that they consider to be important. Participants reported evaluating their progress with respect to both the past and desired targets. However, progress on goals that pertained to security, responsibility, and/or the avoidance of negative outcomes (i.e., were prevention focused) was less likely to be compared with a target than if it did not pertain to these factors. This may be because, as Liberman and Dar (2009) point out, goals that are prevention-focused tend not to have a clear end state. Participants who felt capable of achieving their nominated goal (i.e., had high self-efficacy) reported that it was easier to monitor their progress. This suggests that feeling confident about attaining the goal is positively associated with confidence about being able to assess progress toward that goal (and perhaps feeling confident generally). Finally, we found that participants who framed their goal in quantifiable terms were more likely to report finding it easier to monitor their goal progress and were more likely to compare their current state to a target when assessing their progress. This finding is consistent with the quantity principle described by Josephs et al. (1994), which suggests that people are more likely to judge outcomes based on quantifiable information, particularly when it is easier for them to do so.

## Study 2

One limitation of the approach used in Study 1 is that it required coders to infer how participants construed their goals and how they monitored their progress based on their open ended responses. Therefore, in Study 2 we asked participants to complete an online survey in which they rated aspects of their goals and how they assessed their progress toward them. In addition to the constructs examined in Study 1, Study 2 also examined whether the focal goal’s domain (i.e., whether the goal related to physical development or health, finance, work or studies, or social relationships) was associated with the nature of progress monitoring. Instead of asking participants to evaluate their goal and aspects of progress monitoring on binary scales, we used Likert-type scales to increase the sensitivity of our measures. For instance, instead of asking participants if they assessed their progress with reference to past and/or target comparison points, they rated the frequency with which they assessed their progress with reference to past and/or target comparison points.

### Method

#### Participants

Five hundred and nineteen participants based in the United States were recruited from the crowdsourcing website Amazon MTurk. To ensure that respondents paid sufficient attention to the questions, we included three test questions that simply asked participants to select a specified response. Twenty-seven participants were excluded for answering at least one of the test questions incorrectly, leaving 492 participants for the analysis (*M*_age_ = 33.12, *SD* = 11.26; 232 females, 261 males, 1 of unspecified gender). It took participants approximately 15 min to complete the survey and they were paid $US0.95 for their time.

#### Procedure

Participants completed an online questionnaire using the survey platform Qualtrics, in which they were asked to nominate a goal that they had been working on for at least a week and then to answer questions about that goal.

#### Measures

##### Nature of the goal

Participants were asked to rate: (a) the extent to which their goal was related to security and/or responsibility (i.e., was prevention focused; 1 = my goal is not at all related to security nor responsibility, to 5 = my goal is entirely related to security or responsibility), (b) involved avoiding a negative outcome (1 = my goal exclusively involves avoiding a negative outcome, 5 = my goal exclusively involves attaining a positive outcome), (c) was motivated by intrinsic motivation (1 = I am motivated to work on this goal only because of the outcome I wish to achieve, 5 = I am motivated to work on this goal only because I find working on it enjoyable/satisfying). Participants also rated their level of self-efficacy using a scale adapted from Karoly and Ruehlman (1995). An example item is: “I possess the necessary skills to attain this goal”, (1 = not at all, 5 = extremely).

The two dimensions of prevention focus (i.e., whether the goal was related to security and responsibility and whether the goal was related to the avoidance of losses) were unrelated (*r* = 0.04), and so were retained as separate measures for the analyses (with the measure of the avoidance of negative outcomes reverse-coded for the inferential analyses). Two independent coders rated whether each goal pertained to physical development or health, finance, work or study, or social relationships. There was a high level of inter-rater reliability (free-marginal kappa = 0.99), and the goals were dummy coded with respect to the four goal domains (1 = belonging to the target domain, 0 = not belonging to the target domain) so that they could be entered as predictors into the regression analyses.

##### Nature of monitoring

To encourage participants to start thinking about how they assess their goal progress, they were first asked to rate the extent to which they are aware of how much (or how little) progress they are making (1 = I have no idea if I am on track to achieving my goal, 5 = I know for sure if I am on track to achieving my goal). They were then asked to rate how difficult it was for them to monitor their goal progress, using the same scale as those used in Study 1 (i.e., 1 = very easy and 5 = very difficult). Next, participants rated the frequency with which they assessed their progress by comparing their current situation to: (a) the past and, (b) a target state that they want to reach (or avoid), on a scale from 1 = never to 5 = almost all the time. Finally, participants rated the extent to which they felt that their goal progress could be quantified, for example, in terms of time, weight, amount, number, frequency, etc. (1 = I don’t think about my goal in terms that can be quantified, to 5 = Almost all of how I think about my goal can be quantified).

### Results

#### Nature of Elicited Goals

On average, participants had worked on their nominated goal for 17.22 weeks (*SD* = 46.87). Participants’ goals tended to be more concerned with attaining positive outcomes than preventing negative ones (*M* = 4.02, *SD* = 1.00), and were moderately related to security and responsibility (*M* = 2.73, *SD* = 1.51). On average, participants reported moderate levels of intrinsic motivation for their goal (*M* = 2.34, *SD* = 1.34), and they tended to think about their goal progress in quantifiable terms (*M* = 3.59, *SD* = 1.48). Most of the goals that participants reported on related to physical development or health (36.0%), followed by finances (26.4%), work or studies (18.3%), and relationships (4.3%). Missing data in this and the subsequent study was dealt with using pairwise deletion.

#### Nature of Progress Monitoring

Sign tests indicated that participants more frequently assessed their progress by comparing their current situation to a target state (*M* = 3.81, *SD* = 0.96) than to the past (*M* = 3.46, *SD* = 1.07), Z = -5.68, *p* < 0.001, *r* = 0.26. In general, participants reported that they found it relatively easy to monitor their progress toward the nominated goal (*M* = 2.01, *SD* = 2.05).

#### Factors Associated with the Nature of Progress Monitoring

Three linear regressions were conducted to examine which factors predicted: (a) how frequently participants compared their current state to the past, (b) how frequently they compared their current state to a target, and (c) how difficult they considered monitoring their progress to be. **Table 2** summarizes the findings. Participants more frequently assessed their progress with reference to the past if their goal was related to security and responsibility [*B* = 0.13, *SE* = 0.04, *t*(480) = 2.48, *p* = 0.01, B = 0.09], if they thought about their goal progress in quantifiable terms [*B* = 0.16, *SE* = 0.04, *t*(480) = 3.14, *p* = 0.002, B = 0.11], or if it pertained to social relationships [*B* = 0.10, *SE* = 0.26, *t*(480) = 2.02, *p* = 0.04, B = 0.01]. They were less likely to compare their current situation to the past if their goal was related to financial issues [*B* = -0.21, *SE* = 0.17, *t*(480) = -3.03, *p* = 0.003, B = -0.21], or to work and/or study [*B* = -0.12, *SE* = 0.17, *t*(480) = -2.01, *p* < 0.05, B = -0.12]. Participants more frequently monitored their progress with reference to a target when they thought about their goal progress in quantifiable terms [*B* = 0.13, *SE* = 0.03, *t*(480) = 4.08, *p* < 0.001, B = 0.21].

**Table 2:** Standardized relationships (B coefficients) between predictors (rows) and the nature of progress monitoring (columns) in Study 2.

	Comparison to the past	Comparison to a target	Difficulty of monitoring
Number of weeks spent on goal^a^	–0.04	0.03	0.01
Security and responsibility	0.13ˆ*	0.04	0.06
Avoidance of negative outcomes	0.03	0.06	–0.10ˆ*
Intrinsic motivation	0.08	–0.02	0.06
Quantifiability	0.16ˆ**	0.21ˆ***	–0.17ˆ**
Self-efficacy	0.05	0.08	–0.31ˆ***
Physical development/health goal	0.09	–0.10	0.09
Financial goal	–0.21ˆ**	–0.02	0.05
Work or study goal	–0.12ˆ*	–0.04	0.18ˆ**
Social relationship goal	0.10ˆ*	–0.02	0.06
*R^2^*	0.09	0.09	0.20
*F*	4.92ˆ***	4.43ˆ**	11.80ˆ***

*^∗^*p* < 0.05, ^∗∗^*p* < 0.01, ^∗∗∗^*p* < 0.001. ^a^This variable was log transformed prior to analysis.*

As in Study 1, participants found it easier to monitor their goal progress if they felt confident of achieving their goal [*B* = -0.31, *SE* = -0.02, *t*(480) = -7.24, *p* = 0.001, B = -0.31], and if they thought about their progress in quantifiable terms [*B* = -0.16, *SE* = -0.16, *t*(480) = -3.30, *p* = 0.001, B = -0.16]. Participants considered their goal progress to be more difficult to assess if the goal related to avoiding a negative outcome [*B* = -0.13, *t*(480) = 2.28, *SE* = -0.05, *p* = 0.02, B = -0.10], and was related to work and/or study [*B* = 0.18, *SE* = 0.16, *t*(480) = 3.05, *p* = 0.002, B = 0.18].

### Discussion

Two of the findings from Study 2 are consistent with those of Study 1. First, participants who thought about their goal in quantifiable terms were more likely to monitor their progress toward that goal, and found their progress toward that goal easier to monitor. Second, the more confident that participants felt about achieving their goal, the easier that they felt it was to assess their progress toward that goal. However, unlike Study 1, Study 2 showed that a prevention focus was not associated with less frequent monitoring with reference to a desired target, and that prevention focused goals were considered to be more difficult to monitor (whereas Study 1 did not reveal a significant relationship between prevention focus and monitoring difficulty).

Study 2 also revealed that participants seemed to monitor their progress in different ways for different types of goals. Progress toward goals related to finances and to work and/or study were less likely to be compared with the past. This may be explained by the possibility that people typically try to improve upon their previous financial and work situation, and thus a past state is not the desired outcome. In addition, participants considered goals related to work and/or study to be more difficult to monitor. This may be because monitoring progress toward work and study-related goals often involves requesting feedback, which can be associated with numerous obstacles (Walsh et al., 1985; Harrison et al., 2015).

## Study 3

One limitation of both Studies 1 and 2 was that they used single-item measures to assess factors that might be associated with the nature of progress monitoring. Therefore, Study 3 was designed to provide a conceptual replication of Study 2 by investigating the same relationships in a new sample with multi-item measures of the key constructs.

Another limitation of Study 2 is that it used a bipolar scale to assess motivation, with intrinsic motivation on one end and extrinsic motivation at the other end of the spectrum. Given that intrinsic and extrinsic motivation may not exist on a continuum relative to each other, in Study 3 we improved on this measure by assessing intrinsic motivation on a separate scale.

### Method

#### Participants

Five hundred participants were recruited from the crowdsourcing website Prolific Academic. As before, test questions were randomly placed in the questionnaire to ensure the integrity of data. Fourteen participants were excluded for answering at least one of the test questions incorrectly, one was excluded for answering the questions with respect to multiple goals, instead of with reference to one goal, and two participants were excluded because their responses were incomplete. This left 481 participants for the analysis (*M*_age_ = 27.13, *SD* = 8.05, 150 females, 216 males, 3 of unspecified gender, and 112 who did not give their gender nor age)^1^. Participants took around 15 min to complete the survey and were each paid £1.25 for their time.

#### Procedure

The procedure for Study 3 was the same as in Study 2, except that multiple questions were included to measure each construct.

Questions assessing whether the goal was prevention-focused were adapted from the Regulatory Focus Questionnaire (RFQ; Higgins et al., 2001), which is commonly used to measure individual differences in a prevention versus a promotion focus. Although the RFQ has separate subscales to assess prevention and promotion focus, we only used the items from the prevention-focused subscale in this study. Example questions include “In order to achieve this goal, I sometimes have to cross the line by doing things that some people might not tolerate” (reverse-coded), “What I do to achieve my goal might get on some people’s nerves.” (reverse-coded) “Working on this goal requires me to act in ways that people might find objectionable” (reverse-coded). Internal consistency reliability of this scale was satisfactory (ordinal α = 0.84^2^, Cronbach’s α = 0.79). These questions are designed to measure the extent to which individuals act in a way that conforms to rules and regulations, which is thought to characterize a prevention-focus.

Intrinsic motivation was measured by adapting four items from the Situational Motivation Scale (Guay et al., 2000): “I am working on this goal because I find it interesting to do so/I think that it is pleasant to do so/it is fun/I feel good when doing it.” These questions were all answered on a 5-point Likert scale anchored by 1 = strongly disagree to 5 = strongly agree. The internal consistency reliability of this scale was high (ordinal α = 0.91, Cronbach α = 0.87).

The extent to which participants felt that their progress toward their nominated goal could be quantified was assessed with four questions: “I use a number to express how much progress I have made toward this goal,” “I assess my progress toward this goal in quantifiable terms (for example, in terms of time, weight, frequency, etc.,” “It is difficult for me to express in numerical terms whether or not I am making progress toward this goal” (reverse-coded), and “I try to quantify my progress toward this goal (for example, whether it has changed by a certain amount).” Participants answered on a scale of 1 = strongly disagree to 5 = strongly agree. Internal consistency reliability of this scale was high (ordinal α = 0.85; Cronbach’s α = 0.81).

Levels of self-efficacy were assessed with four questions: “I possess the necessary skills to attain this goal,” “I have the necessary knowledge to reach this goal,” “I have what it takes to reach this goal,” and “I have the ability to reach this goal.” The questions were answered on a scale of 1 = strongly disagree to 5 = strongly agree. Internal consistency reliability of this scale was again satisfactory (ordinal α = 0.88, Cronbach’s α = 0.82).

Two independent coders rated whether each goal related to physical development or health, finance, work or studying, or social relationships. Inter-rater reliability was very high (free-marginal kappa = 0.97).

### Results

#### Nature of Elicited Goals

Participants had spent an average of 25.27 weeks (*SD* = 79.16) striving for their goal. Goals were typically prevention focused (*M* = 3.77, *SD* = 0.95) and intrinsically motivated (*M* = 3.77, *SD* = 0.95). Participants reported relatively high levels of self-efficacy (*M* = 4.26, *SD* = 0.95), and tended to assess their progress in quantifiable terms (*M* = 4.83, *SD* = 0.58). Participants most commonly reported goals related to work or studies (41.0%), followed by goals related to physical development or health (32.9%), finances (11.6%), and relationships (3.5%).

#### Nature of Progress Monitoring

Participants more frequently assessed their progress by comparing their current situation to a target (*M* = 3.83, *SD* = 0.92) than to a past state (*M* = 3.52, *SD* = 1.05), *t*(482) = -5.57, *p* < 0.001, Cohen’s *d* = 0.32, and felt that monitoring their goal progress was moderately easy (*M* = 2.44, *SD* = 1.12).

#### Factors Associated with the Nature of Progress Monitoring

**Table 3** summarizes the findings of regression analyses with the nature of progress monitoring as dependent variables. Participants less frequently monitored their progress with respect to a past state if their goal was prevention focused [*B* = -0.10, *SE* = 0.05, *t*(468) = -2.10, *p* = 0.04, B = -0.11], or was related to finance [*B* = -0.15, *SE* = 0.20, *t*(468) = -2.54, *p* = 0.01, B = -0.50], or work or study [*B* = -0.24, *SE* = 0.16, *t*(468) = -3.25, *p* = 0.001, B = -0.51]. However, they were more likely to monitor their progress with respect to a past state if they thought about their progress in quantifiable terms [*B* = 0.12, *SE* = 0.06, *t*(468) = 2.35, *p* = 0.02, B = 0.14].

**Table 3:** Standardized relationships (B coefficients) between predictors (rows) and the nature of progress monitoring (columns) in Study 3.

	Comparison to the past	Comparison to a target	Difficulty of monitoring
Number of weeks spent on goal^a^	0.02	0.16ˆ**	–0.05
Prevention focus	–0.10ˆ*	0.05	–0.20ˆ***
Intrinsic motivation	0.05	–0.07	0.01
Quantifiability	0.12ˆ*	0.24ˆ***	–0.36ˆ***
Self-efficacy	0.01	0.02	–0.16ˆ***
Physical development/health goal	–0.05	–0.13	0.03
Financial goal	–0.15ˆ*	–0.07	0.10
Work or study goal	–0.24ˆ**	–0.01	0.14ˆ*
Social relationship goal	–0.03	–0.04	0.07
*R^2^*	0.07	0.08	0.25
*F*	4.15ˆ***	4.64ˆ***	16.93ˆ***

*^∗^*p* < 0.05, ^∗∗^*p* < 0.01, ^∗∗∗^*p* < 0.001. ^a^This variable was log transformed prior to analysis.*

Participants more frequently monitored their progress with respect to a target when they had worked on their goal for a long period of time [*B* = 0.16, *SE* = 0.03, *t*(468) = 3.49, *p* = 0.001, B = 0.11] and/or when they thought about their goal progress in quantifiable terms [*B* = 0.24, *SE* = 0.05, *t*(468) = 4.78, *p* < 0.001, B = 0.24].

Participants felt that their progress was easier to assess if their goal was prevention focused [*B* = -0.20, *t*(468) = -4.67, *SE* = 0.05, *p* < 0.001, B = -0.23], if they thought about their goal progress in quantifiable terms [*B* = -0.36, *SE* = 0.06, *t*(468) = -7.86, *p* < 0.001, B = -0.44], and if they had relatively high levels of self-efficacy [*B* = -0.26, *SE* = 0.09, *t*(468) = -3.78, *p* < 0.001, B = -0.34]. Participants found progress toward goals related to work and study [*B* = 0.14, *SE* = 0.15, *t*(468) = 2.08, *p* = 0.04, B = 0.31] more difficult to monitor than goals in other domains.

### Discussion

The findings of Study 3 support those of Studies 1 and 2 in showing that the more that participants thought about their goal progress in quantifiable terms, the more that they monitored their progress, and the easier that they felt monitoring to be. Also consistent with the previous studies, Study 3 showed that participants who were confident that they could achieve the goal also felt confident that they could assess their progress toward that goal. In keeping with Study 2, the results from Study 3 indicated that participants were less likely to assess their progress toward goals related to finance and to work and study with reference to the past, and they found monitoring their progress toward goals related to work and study to be more difficult to assess.

Participants were less likely to monitor their progress with reference to the past if they had worked on their goal for a long, relative to a short, period of time. This finding is consistent with those of Bonezzi et al. (2011), who showed that the further people were away from their initial starting point in goal pursuit (something that is likely when people have been striving for a goal for a long period of time), the less likely they were to assess their progress with reference to the initial starting point.

In contrast to Study 2, however, the results from Study 3 showed that progress toward prevention-focused goals was less likely to be evaluated with respect to the past (Study 2 revealed that progress toward goals related to security and responsibility was *more* likely to be evaluated with respect to the past). One explanation for the difference in findings may be the use of different measures in the two studies. For example, the question used to assess prevention focus in Study 2 asked participants to report the extent to which their goal was related to security and responsibility, whereas the questions used to assess prevention focus in Study 3 asked participants to report the extent to which working on the goal required them to engage in objectionable behavior. In addition, participants in Study 3 considered progress toward goals that were associated with a prevention focus to be easier to assess, but participants in Study 2 considered progress toward goals related to the avoidance of negative outcomes to be harder to assess, suggesting that a prevention focus may have reflected subtly different beliefs in the two studies.

It is also worth noting that it may be difficult to make direct comparisons between the participants recruited for Studies 2 and 3, as there were some differences between the two samples. For instance, participants in Study 3 tended to be younger than those in Study 2 (*M*_age_ = 27.13 in Study 3, and *M*_age_ = 33.12 in Study 2), and they tended to have worked on their goal for longer (*M* = 25.27 weeks), than those in Study 2 (*M* = 17.22 weeks). Participants in Study 3 also tended to report a higher proportion of goals related to work and/or study (41.0%), than participants in Study 2 (18.3%). These differences may account for the differences in results found between the two studies.

## General Discussion

The first aim of the present research was to investigate how people monitor their progress toward goals and identify factors that influence the reference points that people use to assess their goal progress (i.e., past vs. target), and the frequency with which they make these comparisons. Study 1 interviewed participants to explore, in an open-ended, exploratory fashion, whether and how they monitored their progress toward goals that they considered to be important. The main findings were that: (a) progress on goals that pertained to security, responsibility, and/or the avoidance of negative outcomes was less likely to be assessed with respect to a target, (b) participants who had high self-efficacy felt that it was easier to monitor their progress, and (c) participants who framed their goal progress in quantifiable terms found it easier to assess their progress and were more likely to compare their current state to a target when assessing their progress.

Studies 2 and 3 investigated similar relationships using a questionnaire design in which participants rated the nature of a nominated goal, along with whether and how they monitored their progress toward that goal. The findings of these studies supported Study 1 in showing that participants were more likely to assess their progress with respect to a target when they thought about their progress in quantifiable terms, and they also showed that participants were also more likely to monitor their goals with respect to the past for such goals. Also consistent with Study 1 was the finding that participants felt that their progress was easier to assess when progress was thought of in quantifiable terms, and when they felt confident that they would be able to attain the goal.

People may more readily monitor their progress toward goals that they think about in quantifiable terms because people are more influenced by quantifiable information (at the expense of other, more diagnostic information) when this information is readily apparent (Josephs et al., 1994). However, people may not monitor appropriately in such situations. For example, Josephs et al. demonstrated that people’s judgments about the clarity and cogency of their essay writing is influenced by their perception of how much they have written (something that is not usually a good indicator of clarity and/or cogency). Consistent with the idea that people may use non-diagnostic features to judge their goal progress, one participant in Study 1 of the present research stated that “At work, my goals are to do well…I want my boss to think that I’m doing well. I’d like to get good results from my experiments.” (P24). However, when the participant was asked how s/he judged her progress, s/he explained “Well, I suppose the hours I’m working…I don’t really have anything else to judge it by.” In such cases, the tendency to focus on aspects of goal pursuit that are easy to quantify may come at the expense of attending to other information that could be used to assess progress, and/or distract people from putting effort into more relevant aspects of goal pursuit (e.g., trying to improve the quality rather than the quantity of their work). Therefore, in addition to seeking to increase the frequency of monitoring, interventions designed to promote goal attainment might also encourage people to monitor aspects of progress that are likely to inform subsequent efforts at goal striving, as allowing people to focus on their preferred dimensions may not always be the most effective for goal pursuit.

With regards to the effects of prevention focus on monitoring goal progress, Study 1 showed that participants were less likely to compare their current state to a desired target if their goal related to avoiding negative outcomes and/or to security and responsibility. In contrast, Study 2 did not demonstrate a relationship between prevention focus and use of a target to assess progress, but indicated that participants were more likely to compare their current state to a past state if the goal in question related to security and responsibility (i.e., was prevention focused). In contrast, Study 3 revealed that participants were *less* likely to compare their current state to a past state if it was prevention focused, as measured by questions adapted from the RFQ (and also did not find a relationship between prevention focus and the use of a target to assess progress). The different relationships between prevention focus and the nature and extent of progress monitoring across the studies may relate to the fact that prevention focus was operationalized in a multidimensional manner, with at least two of the dimensions appearing to be independent of each other. Future research might seek to disentangle the effects of security, responsibility, and the avoidance of negative outcomes by independently manipulating them, in order to examine how each factor influences monitoring. Nevertheless, taken together, the evidence suggests that people are less likely to monitor their progress toward goals that pertain to the avoidance of negative outcomes than those that pertain to the attainment of positive outcomes. Further studies are, however, needed to determine why this is the case, as this relationship may be specific to certain characteristics of the avoidance goals focused on in the present research. Goals that involve maintaining, rather than changing, the current state (such as the goal to maintain weight) are probably less likely to engender changes in progress compared to goals where people are working toward changing their current state (e.g., trying to lose weight). As such, these types of goals may lead to less frequent monitoring not because they are more difficult to monitor, but because frequent updates on progress would be redundant.

The second aim of the present research was to investigate the factors that are associated with how easy or difficult people find monitoring their goal progress to be. Across the studies, participants found it easier to monitor their progress toward goals that they felt more confident of attaining. However, self-efficacy was not associated with the frequency with which participants monitored their progress. This may suggest either that being confident about achieving the goal makes people feel that their progress is easier to assess (i.e., a type of confirmation bias), and/or that the ease of assessing progress increases peoples’ confidence in their ability to achieve the goal. For example, Schunk (1983) found that children who were directed to monitor the number of subtraction exercises that they completed had higher levels of self-efficacy for this task than children who did not monitor their progress, presumably because increased awareness of their accumulating progress increased their confidence that they would be able to achieve the goal.

### Limitations

The present research employed a multi-method approach to identify factors that influence the nature, likelihood, and difficulty of progress monitoring. This was achieved by combining current theoretical perspectives (e.g., that proposed by Liberman and Dar, 2009) with a relatively naturalistic assessment of how people monitor their progress toward goals in a wide variety of domains. Similar approaches combining top-down (i.e., theory and model-driven) and bottom-up (i.e., driven by the experiences of those involved) approaches have proved effective for investigating research questions in contexts where people may adopt a number of different strategies (e.g., Skinner et al., 2003; Webb et al., 2012). However, one limitation of the approach taken in the present research is that the studies were correlational. Although correlational designs prevent strong inferences about causality, many of the present findings are consistent with previous theory and/or empirical research. For example, Josephs et al. (1994) proposed that people would be more likely focus on quantifiable information when it is readily available. Consistent with this idea, we found that people were more likely to focus on this information it was readily available (i.e., they could use their behavior as a proxy for goal progress). Furthermore, our methods overcome many of the shortcomings that are associated with laboratory-based studies. For example, it is difficult to study the effects of intrinsic motivation in laboratory studies where participants are typically assigned goals that may not be personally meaningful to them. It would also be difficult for experimental studies to capture the range of ways that people monitor their progress toward personal goals in their everyday lives. We hope, however, that our findings will provide the impetus for future research that can examine the factors that influence progress monitoring in more detail and examine potential moderators/mediator of these relations.

## Conclusion

Although many theoretical frameworks for understanding goal pursuit and self-regulation highlight the importance of progress monitoring, they do not describe how people typically monitor their progress, nor do they specify the factors that influence its nature and occurrence. The present research offers new insights into whether and how people monitor their progress toward their personal goals and the factors that influence this process. Our findings suggest that people are more likely to monitor their progress toward goals that involve attaining a positive outcome than those that involve avoiding a negative outcome. We also found that people are less likely to use the past as a comparison standard when assessing their progress toward financial goals and those related to work and study, than goals in other domains (e.g., personal relationships). Our findings suggest that people find it easier to monitor their progress toward goals for which they feel confident of attaining, but harder to monitor their progress toward goals related to work and study. Finally, the findings suggest that people monitor their goal progress with respect to a desired target more frequently when they construe their goal progress in quantifiable terms, and that they find it easier to assess their progress toward such goals. Our hope is that these studies lay the groundwork for further theory-building and research on the antecedents of monitoring goal progress.

## Ethics Statement

This study was carried out in accordance with the recommendations of the British Psychological Society Ethical guidelines, with written informed consent from all subjects. All subjects gave written informed consent in accordance with the Declaration of Helsinki. The protocol was approved by the ‘University of Sheffield’s Department of Psychology Ethics Committee.’

## Author Contributions

BC designed and conducted all the studies in the paper. She also analyzed the data for all the studies, and wrote the article. TW proposed the research question for all studies, and was involved in the design of all the studies. He proofread the paper. YB was involved in the design of all the studies and proofread the paper. CS was involved in the data analysis of the first study, and proofread the paper.

## Conflict of Interest Statement

The authors declare that the research was conducted in the absence of any commercial or financial relationships that could be construed as a potential conflict of interest.

## Footnotes

1 This missing data was due to a temporary technical error with the survey platform which resulted in a subset of the participants not being presented with demographic questions.

2 Because responses were on 5-point Likert scales, we report ordinal α following the recommendation of Gadermann et al. (2012). However, we also report Cronbach’s α for the purposes of comparison.
