# Supplementary material for: Which Factors Are Associated with Monitoring Goal Progress?
Source: Front Psychol. 2017 Mar 24;8:434. doi: 10.3389/fpsyg.2017.00434 (PMC5364185; doi:10.3389/fpsyg.2017.00434)
Supplement: Supplementary file 1 [file Data_Sheet_1.docx]

**Appendix: Nature of the goals nominated by participants in Study 1**

*Goals pertaining to physical development and/or health*

Lose / maintain weight

Improve / maintain fitness

Eat more healthily

Reduce the number of cigarettes smoked

*Financial goals*

Save money / sort out finances

# *Goals pertaining to work or study*

Get a job

Accomplish a particular task at work

Succeed or advance at work

*Goals pertaining to social relationships*

Get into a new relationship / maintain relationship

Meet new people / make friends

Spend more time with partner / family

Support family members / close others

Stay in touch with friends

Be a good mum / wife / daughter

*Goals pertaining to personal development*

Make a difference in the world

Avoid giving a negative impression to other people

Avoid becoming close-minded

Work on reducing stress / staying calm / gaining control over feelings

Be happy / more assertive

Be better with self / resolve psychological issues

Improve personal skills

*Other goals*

Work on, or advance in, hobby

Campaign for marriage equalization

Get daughter to eat with knife and fork

Make sure that daughter is as happy as possible

Improve driving / pass driving test

Prepare for a holiday

Buy / sell a house

Decorate / renovate home

Keep house clean and tidy / do more housework
